# Pharmacological activities of *Xanthosoma sagittifolium* (L.) Schott and *Corchorus olitorius* L. extracts in uterine leiomyoma: evidence from *in vitro* and *in vivo* approaches

**DOI:** 10.3389/fphar.2026.1879860

**Published:** 2026-08-19

**Authors:** Mariam Adeyinka Fafure, Oluwadamilola Grace Adedoyin, Peluola Olujide Ayeni, Akingbolabo Daniel Ogunlakin, Mubo Adeola Sonibare, Rashed N. Herqash, Joe Miantezila Basilua, Abdelaaty A. Shahat

**Affiliations:** 1 Department of Pharmacognosy and Herbal Medicine, Faculty of Pharmaceutical Science, University of Ibadan, Ibadan, Nigeria; 2 Phytomedicine and Drug Discovery Research Laboratory (PDD-RL), Biochemistry Programme, Bowen University, Iwo, Nigeria; 3 Pan African University of Life and Earth Sciences Institute (Including Health and Agriculture), Ibadan, Oyo, Nigeria; 4 Center for Research on Medicinal, Aromatic, and Poisonous Plants, DSR, King Saud University, Riyadh, Saudi Arabia; 5 Department of Biostatistics and Mathematics, Faculty of Pharmacy, Paris Cité University, Paris, France

**Keywords:** Corchorus olitorius, monosodium glutamate, oxidative stress, uterine leiomyoma, Xanthosoma sagittifolium

## Abstract

**Introduction:**

Uterine fibroids (leiomyomas) are benign uterine tumors common in reproductive years. Metabolic syndrome—marked by obesity, insulin resistance, hypertension, and dyslipidemia—has been associated with increased fibroid risk. Two Nigerian plants, *Xanthosoma sagittifolium* (cocoyam) and *Corchorus olitorius* (jute mallow), are traditionally used to support women’s reproductive health, though their antifibrotic potential remains underexplored. This study investigated their protective properties against fibroid development using *in vitro* and *in vivo* models.

**Methods:**

Fresh corms of *X. sagittifolium* and leaves of *C. olitorius* were collected, authenticated, air-dried, pulverized, and extracted with 70% ethanol. Antioxidant activities were assessed using DPPH, ABTS, FRAP, nitric oxide scavenging, and Fe^2+^ chelation assays. Enzyme inhibitory activities were tested against α-amylase, α-glucosidase, and acetylcholinesterase. Uterine leiomyoma was induced in female albino rats *via* oral monosodium glutamate (MSG). Animals were grouped into control, MSG-induced, standard drug-treated, and extract-treated sets. Blood samples were analyzed for hormones, while ovaries and the cervix were examined histologically. Phytochemical profiling was performed using HPLC.

**Results and Discussion:**

*C. olitorius* leaf extract showed the strongest antioxidant activity, with a DPPH IC_50_ of 0.132 ± 0.005 μg/mL. ABTS values ranged from 37.50 to 42.80 mmol TEAC/100 g, while FRAP values were 0.0700–0.1500 mg AAE/g. Fe^2+^ chelation IC_50_ values ranged from 0.143 to 0.188 μg/mL, with notable nitric oxide scavenging. Enzyme assays revealed significant α-amylase inhibition by *C. olitorius* (IC_50_ 0.143 μg/mL), while α-glucosidase inhibition was strongest in *X. sagittifolium* corm extract. Acetylcholinesterase inhibition was highest in *C. olitorius* (IC_50_ 3.567 μg/mL). HPLC profiling showed distinct phytochemical compositions across extracts. *In vivo*, MSG disrupted reproductive hormones and tissue architecture, while plant extracts restored hormonal balance and improved histology, comparable to the standard drug. These findings support antioxidant, enzyme inhibitory, and endocrine-modulating properties but have not yet established therapeutic efficacy against uterine leiomyoma in humans. Therefore, further studies on direct uterine tissue evaluation and fibrosis-specific biomarker analysis are hereby recommended.

## 1 Introduction

Uterine fibroids, also called *leiomyomas or myomas*, are benign, monoclonal tumors of smooth muscle cells of the myometrium and are the most common gynecological tumors in women (Pinto, 2024). The relationship between uterine fibroids and diabetes within the context of metabolic syndrome is intricate. Metabolic syndrome, which includes obesity, insulin resistance, hypertension, and dyslipidemia, has been shown to increase the risk of fibroid development (Qin et al., 2021; Ara et al., 2024; Mitro et al., 2025). Insulin resistance and hyperinsulinemia stimulate growth factors that promote fibroid proliferation, further fueling fibroid growth (Olusi et al., 2024). Some studies reported that women with diabetes could develop fibroids (Aninye et al., 2025; Ogunlakin et al., 2025a); however, insulin resistance, a hallmark of both diabetes and metabolic syndrome, remains a significant contributor to fibroid pathogenesis (Zhao et al., 2023; Sun et al., 2025). Fibroids can cause dysmenorrhea and/or pelvic pain, resulting in dyspareunia (Ferrero et al., 2006; Ertunc et al., 2009). Fibroids trigger heavy and/or prolonged menstrual bleeding; this, combined with dyspareunia, could negatively influence the frequency of intercourse, resulting in a reduced chance of achieving successful pregnancy (Moshesh et al., 2014). Fibroid-related symptoms include abnormal menstrual bleeding and especially heavy menstrual bleeding, pelvic pain, pressure complaints leading to urinary or gastrointestinal symptoms, sexual dysfunction, and infertility. Some women might not exhibit any symptoms, but others suffer from severe discomfort and consequences that require medical or surgical treatment (Micić et al., 2024).

By the age of 50, up to 70%–80% of women worldwide suffer from fibroids, with women of African heritage having the highest frequency (Sefah et al., 2023). According to recent studies, 20%–30% of Nigerian women suffer from fibroids; in tertiary hospitals, some regional studies have reported prevalence rates as high as 27.8% in Nigeria (Haile et al., 2025; Lawal et al., 2019; Igbodike et al., 2024). Current treatment of uterine fibroids involves invasive and noninvasive methods like myomectomy, hysterectomy, hormone replacement therapy, and uterine artery embolization. Given the risks, side effects associated with these methods, and healthcare costs, there is an increased demand to find alternative and cheap means for the treatment and management of numerous disease conditions. A better treatment alternative with lower risk and negative effects on the individual, as well as one that is highly accessible and comparatively less expensive, is required due to the orthodox medications’ negative effects and transient symptomatic cure (Ajayi et al., 2025; Ogunlakin et al., 2025b). This includes the use of food-based medicinal plants (nutraceuticals) (Cabanlit et al., 2024; Rahm, 2025).

*Xanthosoma sagittifolium* (L.) Schott (Araceae), commonly known as cocoyam or tannia (Falade and Okafor, 2015), of the family Araceae, is an important edible root crop widely cultivated and consumed in tropical and subtropical regions, including Nigeria. Cocoyam is valued both for its corms and leaves. The corms are consumed boiled or pounded and used in traditional soups and porridges, while the leaves are used as vegetables in various local dishes such as *ekpang nkukwo* and *ofe ede*. Several ethnomedical uses of cocoyam have been recorded in Nigerian traditional medicine, including the treatment of diarrhea and skin irritations and as a food for convalescents due to its easily digestible starch (Ukom et al., 2014; Oridupa et al., 2017). *Xanthosoma sagittifolium* is known to possess various biological properties, including anti-inflammatory, antioxidant, antimicrobial, and anti-ulcer activities. Its mucilage content contributes to its soothing effect on the gastrointestinal tract, while its leaves are rich in dietary fiber, vitamins (notably vitamins A and C), and minerals such as calcium and iron (Boakye et al., 2018). Furthermore, *Corchorus olitorius*, belonging to the family Malvaceae, is an annual herbaceous plant with a thin stem. It can be found in all tropical and subtropical regions because it is used as a popular leafy soup (Kundu et al., 2012). It is considered a nutritious vegetable due to its high content of vitamins, minerals, and phenolic compounds (Giro and Ferrante, 2016). *C. olitorius* L. (Malvaceae**)** is a stiff and fibrous annual herb that grows up to 4 m tall; it is also known as bush okra, wild okra, Jew’s mallow, tossa jute, long-fruited jute, Meloukia, Moroheia, Moroheiya, Mulukhiyah (and other spelling variations), nalta jute, and Tasso (Islam et al., 2013), and it is locally known in Nigeria as ewedu. It is utilized as an herbal remedy for fevers, chronic cystitis, dysentery, and aches, in addition to its cooking purposes (Al-Yousef et al., 2017). In some parts of Nigeria, the leaf decoction is used for the treatment of iron deficiency as well as folic acid deficiency, in addition to the use of leaves in ascites, dysuria, pectoral pain, and female infertility (Fasinmirin and Olufayo, 2009). *C. olitorius* is known to have a wide spectrum of interesting bioactivities, including antioxidant, hepatoprotective, anti-inflammatory, immunostimulant, antitumor, antimicrobial, antidiabetic, analgesic, and cardioprotective activities, in addition to the wound-healing properties and its role in the prevention of obesity (Abdel-razek et al., 2022). Despite their widespread use, there is limited experimental evidence on the antifibrotic effects of *Corchorus olitorius* L. and *Xanthosoma sagittifolium* on uterine leiomyoma. This study is therefore designed to investigate the antifibrotic effects of selected indigenous medicinal plants, *Xanthosoma sagittifolium* and *Cochorus olitorius* L., using an MSG-induced uterine leiomyoma model in albino rats.

## 2 Materials and methods

### 2.1 Chemicals, reagents, drugs and solvents

Analytical-grade chemicals and reagents were employed for the study. The following items were purchased from Sigma Aldrich and Chemie GmH (Steinheim, Germany): quercetin, chlorogenic acid, scopolamine, sodium dihydrogen, disodium hydrogen, dinitro salicylic acid (DNSA), acetylcholinesterase (AChE), butyrylcholinesterase (BChE), benzene, ascorbic acid, tris HCL, thiobarbituric acid (TBA), potassium ferricyanide (KFeCN), trichloroacetic acid (TCA), iron sulphate (FeSO_4_), and hydrogen peroxide (H_2_O_2_), 1’1-diphenyl-2-picryl hydrazine (DPPH), glacial acetic acid, 5,5’-dithiobis-(2-nitrobenzoic acid) (DTNB), semicarbazide Other solvent and drugs used are ethanol, methanol, distilled water, clomiphene citrate, monosodium glutamate.

### 2.2 Preparation of plant extracts

#### 2.2.1 Plant collection and authentication

*Corchorus olitorius* L. and corms of *Xanthosoma sagittifolium* were obtained from Bodija market in Ibadan, Nigeria. The plants were identified and authenticated at the Forest Herbarium Ibadan (FHI), Forest Research Institute of Nigeria (FRIN), where voucher numbers were attained.

#### 2.2.2 Plant preparation

The plant materials were properly cleaned to avoid contamination and air-dried for 2 weeks. They were further oven-dried at 40 °C; the dried samples were pulverized into coarse powder using a mortar and pestle.

#### 2.2.3 Extraction methods

The plant samples were extracted using 70% ethanol and 30% distilled water. 200 g of the pulverized samples of *Corchorus olitorius* leaves, corms of *Xanthosoma sagittifolium,* and corm peel of *Xanthosoma sagittifolium* were macerated in 70% ethanol with regular stirring. The mixture was filtered using filter paper, and the extract obtained was concentrated with a rotary evaporator and dried on a water bath at 40 °C.

### 2.3 *In vitro* antioxidant assay

#### 2.3.1 Determination of ferric reducing property (FRAP)

The ferric reducing property was determined using the method of Oboh et al. (2016). The reducing property of the *Corchorus olitorius* leaves, corms of *Xanthosoma sagittifolium,* and corm peel of *Xanthosoma sagittifolium* was determined by assessing the ability of the extracts to reduce FeCl_3_ solution as described by Oyaizu (1986). A 2.5 mL aliquot was mixed with 2.5 mL of 200 mM sodium phosphate buffer (pH 6.6) and 2.5 mL of 1% potassium ferricyanide. The mixture was incubated at 50 °C for 20 min, and then 2.5 mL of 10% trichloroacetic acid was added. This mixture was centrifuged at 801 *g* for 10 min 5 mL of the supernatant was mixed with an equal volume of water and 1 mL of 0.1% ferric chloride. The absorbance was measured at 700 nm, and the percentage ferric reducing power was subsequently calculated using ascorbic acid equivalent.

#### 2.3.2 Determination of Fe^2+^ chelating ability

The ability of *C. olitorius* leaves, corms of *X. sagittifolium,* and corm peel of *X. sagittifolium* to chelate Fe^2+^ was assessed using the method described by Oboh et al. (2007) *.* The Fe^2+^ chelating ability of *C. olitorius* leaves, corms of *X. sagittifolium,* and corm peel of *X. sagittifolium* was determined using a modified method of Oboh et al. (2007). Freshly prepared 500 μM FeSO_4_ (150 μL) was added to a reaction mixture containing 168 μL of 0.1 M Tris-HCl (pH 7.4), 218 μL saline, and the samples (*C. olitorius* leaves, corms of *X. sagittifolium,* and corm peel of *X. sagittifolium*). The reaction mixture was incubated for 5 min before the addition of 13 μL of 0.25% 1,10-phenanthroline (w/v). The absorbance was subsequently measured at 510 nm in a spectrophotometer. The Fe^2+^ chelating ability was subsequently calculated.

$$\%\text{ Inhibition}=\left[\frac{\text{absorbance}\;\text{of}\;\text{reference}-\text{absorbance}\;\text{of}\;\text{sample}}{\text{absorbance}\;\text{of}\;\text{reference}}\right]\times 100$$

#### 2.3.3 Nitric oxide scavenging ability

The Griess reagent method was used to carry out the nitric oxide scavenging assay (Ogunlakin et al., 2024). The addition of 1 mL of each of the samples of varied strengths was mixed with 0.3 mL of sodium nitroprusside (5 mM). After that, the test tubes were incubated for 150 min at 25 ^o^C. After 150 min, 0.5 mL of Griess reagent (used after 12 h of preparation; an equivalent amount of 0.01% naphthyl ethylenediamine and 1% sulfanilamide was added to 5% ortho-phosphoric acid in distilled water. At 546 nm, the absorbance was measured.

### 2.4 *In vitro* enzyme inhibitory assays

#### 2.4.1 Acetylcholinesterase (AChE) inhibition assays

Acetylcholinesterase (AChE) inhibitory action of the polyphenolic compounds (*C. olitorius* leaves, corms of *X. sagittifolium,* and corm peel of *X. sagittifolium*) was measured using a modified colorimetric method (Perry et al., 2000). The reaction mixture consisted of 200 μL of brain AChE solution (EC 3.1.1.7) in 0.1 M phosphate buffer, pH 8.0, 100 mL of 3.3 mM 5,5’-dithio-bis (2-nitrobenzoic acid) in 0.1 M phosphate buffer solution, pH 7.0, containing NaHCO_3_ (6 mM), and test drug (0.1–0.4 mM). AChE activity was determined by UV spectrophotometry from the changes in absorbance at 412 nm for 3.0 min at 25 °C using acetylthiocholine iodide (100 mL of 0.05 mM water solution) as the substrate after 20 min at 25 °C. The calculation of the % inhibition was used to express the AChE inhibitory actions of the polyphenolic compounds.

$$\%\text{ Inhibition}=\left[\frac{\text{absorbance}\;\text{of}\;\text{reference}-\text{absorbance}\;\text{of}\;\text{sample}}{\text{absorbance}\;\text{of}\;\text{reference}}\right]\times 100$$

#### 2.4.2 α-amylase inhibition assay

The α-amylase inhibition assay was carried out using the procedure of Ogunlakin et al. (2025a). Appropriate *C. olitorius* leaves, corms of *X. sagittifolium,* and corm peel of *X. sagittifolium* dilution (0–200 μL) and 500 μL of 0.02 M sodium phosphate buffer (pH 6.9 with 0.006 M NaCl) containing porcine pancreatic α-amylase (0.5 mg/mL) were incubated at 25 °C for 10 min. Then, 500 μL of 1% starch solution in 0.02 M sodium phosphate buffer (pH 6.9 with 0.006 M NaCl) was added to each tube. The reaction mixtures were incubated at 25 °C for 10 min and stopped with 1.0 mL of dinitrosalicylic acid colour reagent. Thereafter, the mixture was incubated in a boiling water bath for 5 min and cooled to room temperature. The reaction mixture was then diluted by adding 10 mL of distilled water, and absorbance was measured at 540 nm. The α-amylase inhibitory activity was expressed as percentage inhibition.

$$\%\text{ Inhibition}=\left[\frac{\text{absorbance}\;\text{of}\;\text{reference}-\text{absorbance}\;\text{of}\;\text{sample}}{\text{absorbance}\;\text{of}\;\text{reference}}\right]\times 100$$

#### 2.4.3 α-glucosidase inhibition assay

The α-glucosidase inhibition assay was carried out using the procedure of Ogunlakin et al. (2025a). Briefly, appropriate dilution of the phenolic extracts (0–200 μL) and 100 μL of α-glucosidase solution in 0.1 M phosphate buffer (pH 6.9) was incubated at 25 °C for 10 min. Then, 50 μL of 5 mM *p*-nitrophenyl-α-*D*-glucopyranoside solution in 0.1 M phosphate buffer (pH 6.9) was added. The mixtures were incubated at 25 °C for 5 min before reading the absorbance at 405 nm in the spectrophotometer. The α-glucosidase inhibitory activity was expressed as percentage inhibition.

$$\%\text{ Inhibition}=\left[\frac{\text{absorbance}\;\text{of}\;\text{reference}-\text{absorbance}\;\text{of}\;\text{sample}}{\text{absorbance}\;\text{of}\;\text{reference}}\right]\times 100$$

### 2.5 *In vivo* assays

#### 2.5.1 Experimental animals

Healthy female albino rats weighing 170–240 g were obtained from the animal house in Ogbomoso, Oyo State. They were taken to the experimental study section of the animal house at the University of Ibadan for 1 week before the study to make them adapt to the new environment. They were grouped into 5 groups and were housed 5 animals per cage, with wood shavings as the bedding material and a wire screen top. The cages were well ventilated and kept at room temperature and relative humidity, with a natural light-dark cycle. The animals were fed with commercial pellet rat feed and clean water; good hygiene was also maintained by cleaning the cages and changing wood shavings every 2 days.

#### 2.5.2 Dosing of experimental animals

Prior to the commencement of this research, ethical approval with approval number BUI/BCH/PHARM-UI/01/25/02 was obtained from Bowen University, Iwo, Osun State. The study was adapted from Ogunlakin et al. (2025a), with doses of monosodium glutamate (MSG) administered at 800 mg/kg in this study. Oral gavage was used to dose albino rats (Ogunlakin et al., 2025a); dosing was done once daily over the experimental period at a volume of 1 mL per kg per body weight. Individual dose volume was calculated based on the animal’s most recent body weight; the oral route of administration was used because it is the intended human exposure route.

#### 2.5.3 Experimental procedure

The rats were randomly assigned into five groups, with five rats in each group, as presented in Table 1. Group A served as the normal control and received distilled water only (did not receive any MSG (Vedan Limited) or any extract). Uterine leiomyoma was induced in Groups B to E by oral administration of monosodium glutamate (MSG) at a dose of 800 mg/kg body weight daily for a period of 2 weeks. Following successful induction, Group B received clomiphene citrate (1 mg/kg) as the standard treatment, while Groups C, D, and E received *C. olitorius* leaf extract, *X. sagittifolium* corm extract, and *X. sagittifolium* peel extract, respectively, each at a dose of 100 mg/kg body weight. All treatments were administered orally once daily for an additional 2-week period. At the end of the experimental period, the animals were euthanized by cervical dislocation, and blood samples were collected for the determination of plasma follicle-stimulating hormone (FSH), luteinizing hormone (LH), and estradiol levels. Thereafter, the uterus and cervix were excised, fixed in 10% neutral buffered formalin, and processed for histopathological examination.

**TABLE 1:** Grouping of the animals and dosage of treatments.

Groups	Dosage and administration	Group description
A	Distilled water only	Control
B	800 mg/kg MSG + 1 mg/kg clomiphene citrate	MSG + clomiphene citrate
C	800 mg/kg MSG + 100 mg/kg *C. olitorius*	MSG + *C. olitorius*
D	800 mg/kg MSG + 100 mg/kg *X. sagittifolium* corm	MSG + *X. sagittifolium* corm
E	800 mg/kg MSG + 100 mg/kg *sagittifolium* peel	MSG + *sagittifolium* peel

### 2.6 Hormonal assays

#### 2.6.1 Luteinizing hormone assay

The serum concentration of luteinizing hormone (LH) was quantified using a solid-phase ELISA method (Calbiotech Inc., LH231F). The test was based on a sandwich enzyme immunoassay, where LH in the sample binds to microwells pre-coated with streptavidin. A conjugate reagent was added, followed by a substrate (TMB). After incubation and washing, the color intensity was measured spectrophotometrically at 450 nm.

#### 2.6.2 Follicle-stimulating hormone assay

The level of FSH was measured using a direct sandwich ELISA (Calbiotech Inc., FS046F) and carried out following the manufacturer’s procedure. The assay involved adding the sample and anti-FSH-HRP conjugate to a well coated with monoclonal anti-FSH antibodies. Following incubation, unbound proteins were washed off, and the substrate reaction was allowed to develop color. Absorbance was measured at 450 nm.

#### 2.6.3 Estradiol assay

Serum estradiol level (E2) was determined following the procedure of the manufacturer and using a competitive binding ELISA from Calbiotech Inc., ES380S procedure. The assay involved incubating the sample with anti-E2 antibody and enzyme conjugate. The enzyme-linked antigen competes with endogenous estradiol for the limited binding sites on the antibody. The reaction was stopped with an acid solution, and absorbance will be read at 450 nm. The intensity of color was inversely proportional to the estradiol concentration.

#### 2.6.4 Ovarian and cervical histology

The ovaries and cervixes were examined using a standard procedure. The sections were removed with clean, labeled slides from a water bath that had been heated to 55 °C, dehydrated on a hotplate for an hour at 60 °C, and then inspected under a light microscope with ×40 objectives.

### 2.7 Phytochemical analysis

#### 2.7.1 Determination of 1,1-diphenyl-2-picrylhydrazyl (DPPH) radical scavenging ability

Gyamfi et al. (1999) procedure was used to evaluate the ability of *C. olitorius* leaves, corms of *X. sagittifolium,* and corm peel of *X. sagittifolium* to scavenge free radicals from the 1’1-diphenyl-2-picrylhydrazyl (DPPH) free radicals. One milliliter (1 mL) of each of the plant extracts was diluted appropriately, and 0.4 mM DPPH radicals were added to 1 mL of methanolic solution. After 30 min in the dark, the absorbance value of the mixture was read at 516 nm. Then 2 mL of DPPH solution was used for the control, without the test samples. This was followed by a comparison of the DPPH free radical scavenging ability of the samples to control.

$$\%\text{ Inhibition}=\left[\frac{\text{absorbance}\;\text{of}\;\text{reference}-\text{absorbance}\;\text{of}\;\text{sample}}{\text{absorbance}\;\text{of}\;\text{reference}}\right]\times 100$$

#### 2.7.2 Determination of ABTS radical scavenging activity

The ABTS radical scavenging activity was determined using the modified method of Oboh et al. (2016). The ABTS radical cation (ABTS•^+^) was generated by reacting 7 mM ABTS solution with 2.45 mM potassium persulfate and allowing the mixture to stand in the dark at room temperature for 12–16 h. The resulting ABTS•^+^ solution was diluted with distilled water to obtain an absorbance of 0.70 ± 0.02 at 734 nm. An aliquot of the extracts of *Corchorus olitorius* leaves, corms of *Xanthosoma sagittifolium*, and corm peel of *Xanthosoma sagittifolium* (0.2 mL) was mixed with 2.0 mL of the diluted ABTS•^+^ solution. The reaction mixture was incubated at room temperature in the dark for 15 min. The absorbance was then measured at 734 nm against a blank. Trolox was used as the reference standard, and ABTS scavenging activity was expressed as percentage inhibition or as Trolox equivalents.

#### 2.7.3 HPLC analysis

To identify the phytochemicals in the extracts, High-Performance Liquid Chromatography (HPLC) analysis was conducted using the protocol outlined by Olanrewaju et al. (2024) and Ogunlakin et al. (2025a). With a few modifications, a reverse-phase C18 column (250 mm × 4.6 mm, 5 µm particle size) kept at a steady temperature of 30 °C was used for the analysis. Solvent A (water with 2% acetic acid) and solvent B (methanol) made up the mobile phase. They were applied in a gradient elution profile, beginning with 5% methanol for the first 2 min and increasing the concentration of methanol every 10 minutes for the next ten to 60 min. Each sample had an injection volume of 20 μL, and the flow rate was set at 1.0 mL/min. A UV detector with a wavelength of 254 nm, which is appropriate for tracking a wide variety of phytochemicals, was used to detect the eluted molecules. Standard compounds for the presence of phenolic and flavonoid compounds in the extract included cinnamic acid, rutin, myricetin, gallic acid, caffeic acid, syringic acid, vanillin, p-coumaric acid, ferulic acid, ellagic acid, benzoic acid, catechol, p-hydroxybenzoic acid, vanillic acid, o-coumaric acid, salicylic acid, quercetin, naringenin, kaempferol, and apigenin.

### 2.8 Statistical analysis

Values were displayed as Mean ± Standard deviation (SD). Data were analyzed using one-way analysis of variance (ANOVA), and group means were compared using Dunnett’s Multiple Comparison and Bonferroni tests using GraphPad Prism version 5.01 for Windows, GraphPad Software, San Diego, California, USA. P values that were less than 0.05 were deemed significant.

## 3 Results

### 3.1 Plant authentication and extraction

These plants were collected and authenticated at the Forest Herbarium Ibadan (FHI), Forest Research Institute of Nigeria (FRIN), Jericho, Ibadan. The voucher numbers were assigned, and the voucher specimens were deposited at the herbarium. After extraction, the percentage yield of the plant extract was obtained from the powdered plant. With *Corchorus olitorius* having the highest yield of 6.2% (Table 2).

**TABLE 2:** Percentage yield of the plant extracts.

Plants	Solvent used	Weight of powdered material (g)	Weight of extract (g)	%Yield
*C. olitorius*	70% ethanol	200	12.425	6.2
*X. sagittifolium* corm			7.425	3.7
*X. sagittifolium* peel			6.131	3

### 3.2 *In vitro* antioxidant activity

**FIGURE 1:**
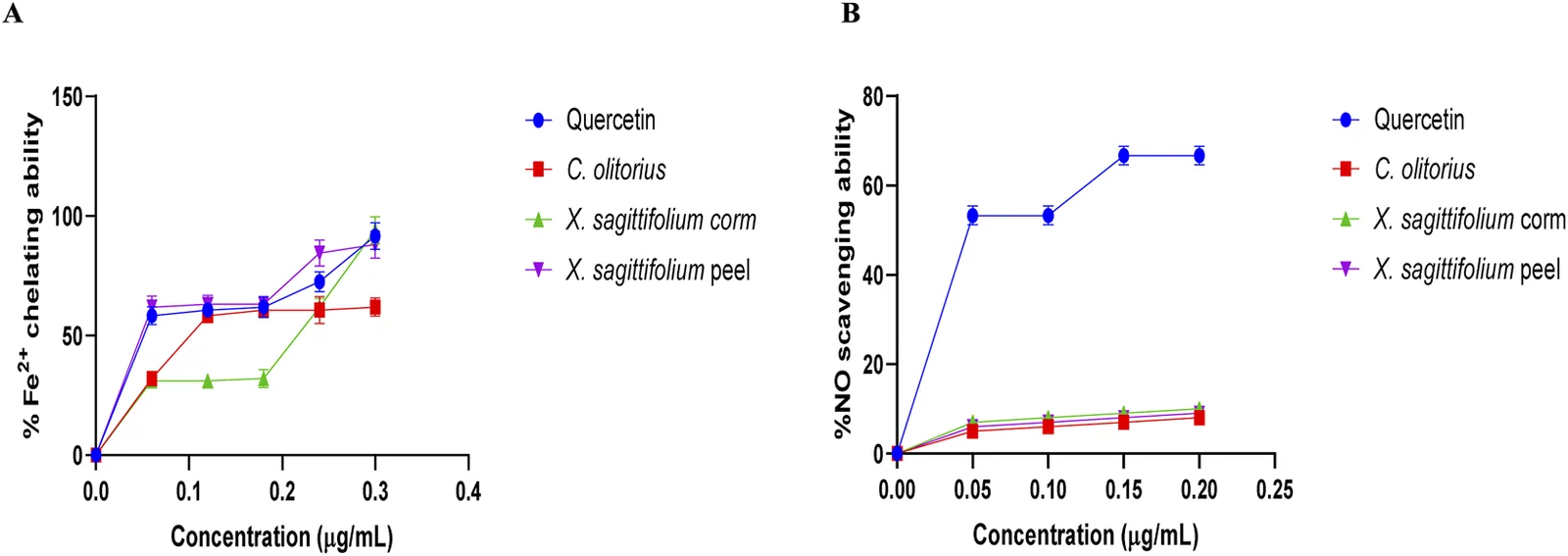
Percentage **(A)** Fe^2+^ chelating, and **(B)** NO radical scavenging abilities of *C. olitorius*, *X. sagittifolium*, *X. sagittifolium* peel and Quercetin (Standard drug/positive control).

**FIGURE 2:**
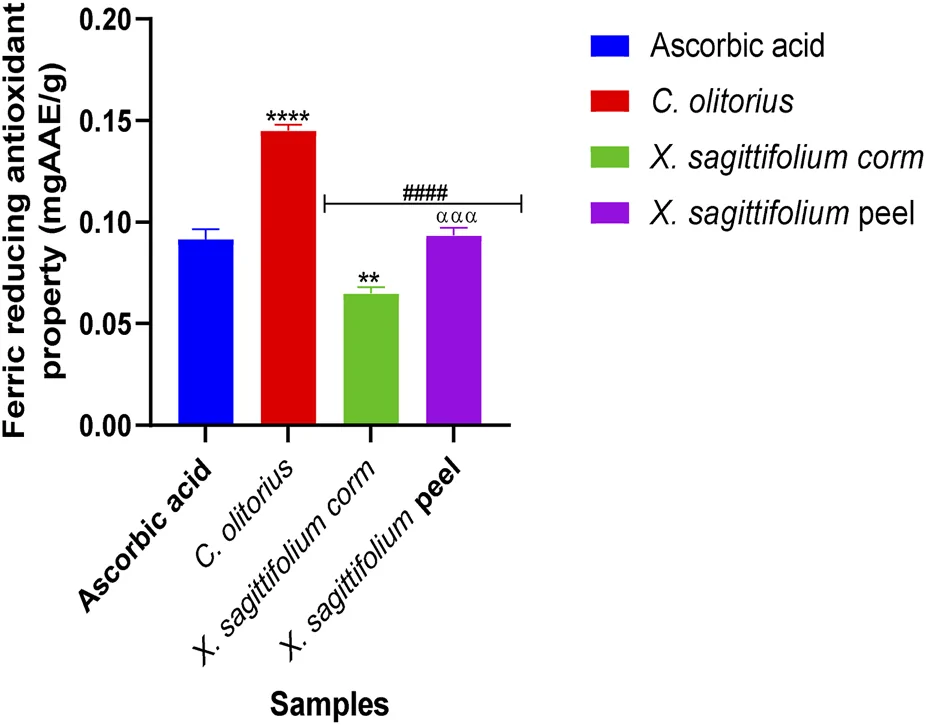
Ferric Reducing Antioxidant Properties of *C. olitorus*, *X. sagittifolium* corm, *X. sagittifolium* peel and Trolox (standard). Bars represent ± SD (n = 3). Values are statistically different at ****p < 0.001 vs. Trolox, ****p < 0.001, **p < 0.01 vs. Ascorbic acid, ^####^ *p <* 0.001 Vs C. *olitorus*, ^ααα^ *p <* 0.001 Vs. *X. sagittifolium* corm.

The Fe^2+^ chelating activities presented in Figure 1A and Table 3 showed that *X. sagittifolium* peel exhibited stronger metal chelating ability than *C. olitorius* and *X. sagittifolium* flesh, with chelating activity comparable to the reference antioxidant. Both *C. olitorius* and *X. sagittifolium* flesh demonstrated similar but lower chelating effectiveness. The nitric oxide scavenging activities depicted in Figure 1B (Table 3) showed that all plant extracts exhibited weaker nitric oxide scavenging ability compared with the standard. The ferric reducing antioxidant power results shown in Figure 2 revealed that *C. olitorius* possessed the highest reducing ability among the plant extracts. *X. sagittifolium* peel demonstrated reducing power comparable to the standard, whereas the flesh extract showed the lowest reducing capacity.

**TABLE 3:** IC_50_ values of the antioxidant potentials of the selected extracts.

Samples	IC50 (mg/mL)	
	Fe Chelation assay	NO scavenging assay
C. olitorus	0.187 ± 0.003^b^	0.833 ± 0.100^b^
X. sagittifolium corm	0.188 ± 0.005^b^	0.833 ± 0.100^b^
X. sagittifolium peel	0.143 ± 0.005^a^	0.937 ± 0.100^b^
Standard(s)	0.148 ± 0.004^a^ (Q)	0.119 ± 0.040^a^ (Q)

Values represent means of triplicate. Values with the same alphabet along the same column are not significantly different (p > 0.05). Control = Quercetin (Q).

### 3.3 Enzymes inhibitory potentials

**FIGURE 3:**
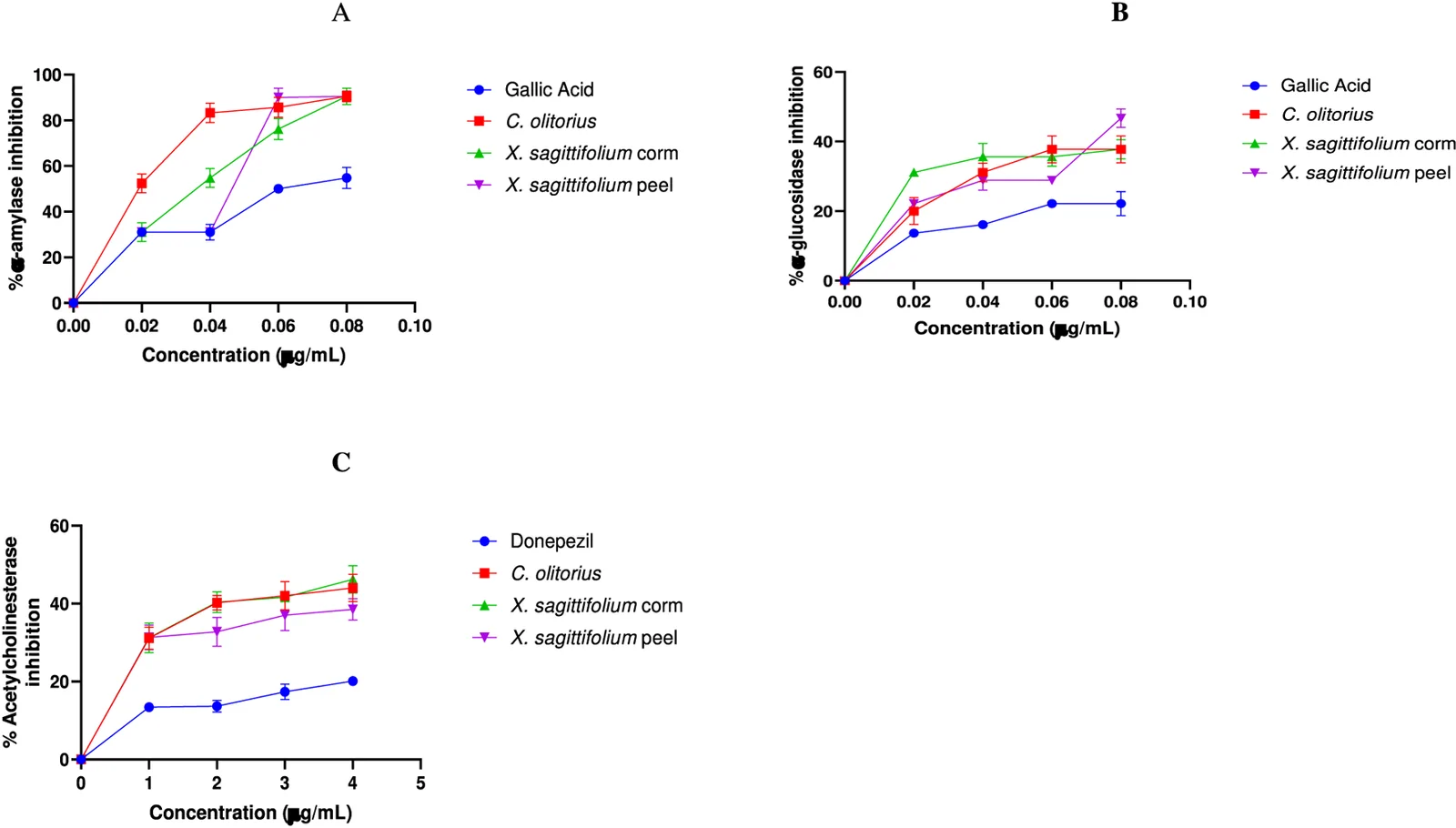
Percentages **(A)** α-amylase Inhibitory, **(B)** α-glucosidase inhibitory, and **(C)** Acetylcholinesterase (AChE) inhibitory abilities of *C. olitorius*, *X. sagittifolium* corm, *X. sagittifolium* peel. Gallic acid and donepezil as control.

The α-amylase inhibitory activities shown in Figure 3A and Table 4 indicate that *C. olitorius* exhibited stronger inhibitory activity than *X. sagittifolium* corm, while both extracts showed greater inhibition than the peel extract. The peel extract displayed inhibitory activity comparable to the standard control. The α-glucosidase inhibitory activities presented in Figure 3B revealed that *X. sagittifolium* flesh exhibited the strongest inhibition among the plant extracts, followed closely by the peel and *C. olitorius*, all of which demonstrated greater inhibitory activity than the standard. The acetylcholinesterase inhibitory activities illustrated in Figure 3C showed that *C. olitorius* and *X. sagittifolium* flesh exhibited stronger inhibition than the peel extract. All plant extracts demonstrated greater acetylcholinesterase inhibitory activity than the reference drug.

**TABLE 4:** IC_50_ values of the enzyme inhibitory activities of the selected extracts.

Samples	IC_50_ (mg/mL)		
	α-amylase inhibitory assay	α-glucosidase inhibitory assay	Acetylcholinesterase inhibitory assay
*C. olitorus*	0.036 ± 0.005^b^	0.087 ± 0.003^b^	3.567 ± 0.020^b^
*X. sagittifolium* corm	0.041 ± 0.005^b^	0.083 ± 0.003^b^	3.567 ± 0.020^b^
*X. sagittifolium* peel	0.066 ± 0.005^a^	0.085 ± 0.003^b^	4.143 ± 0.050^c^
Standard(s)	0.069 ± 0.005^a (GA)^	0.148 ± 0.005^a (GA)^	8.645 ± 0.050^a (D)^

Values represent means of triplicate. Values with the same alphabet along the same column are not significantly different (p > 0.05). Controls: Gallic acid (GA) and Donepezil (D).

#### 3.3.1 *In vivo* hormonal analysis

**FIGURE 4:**
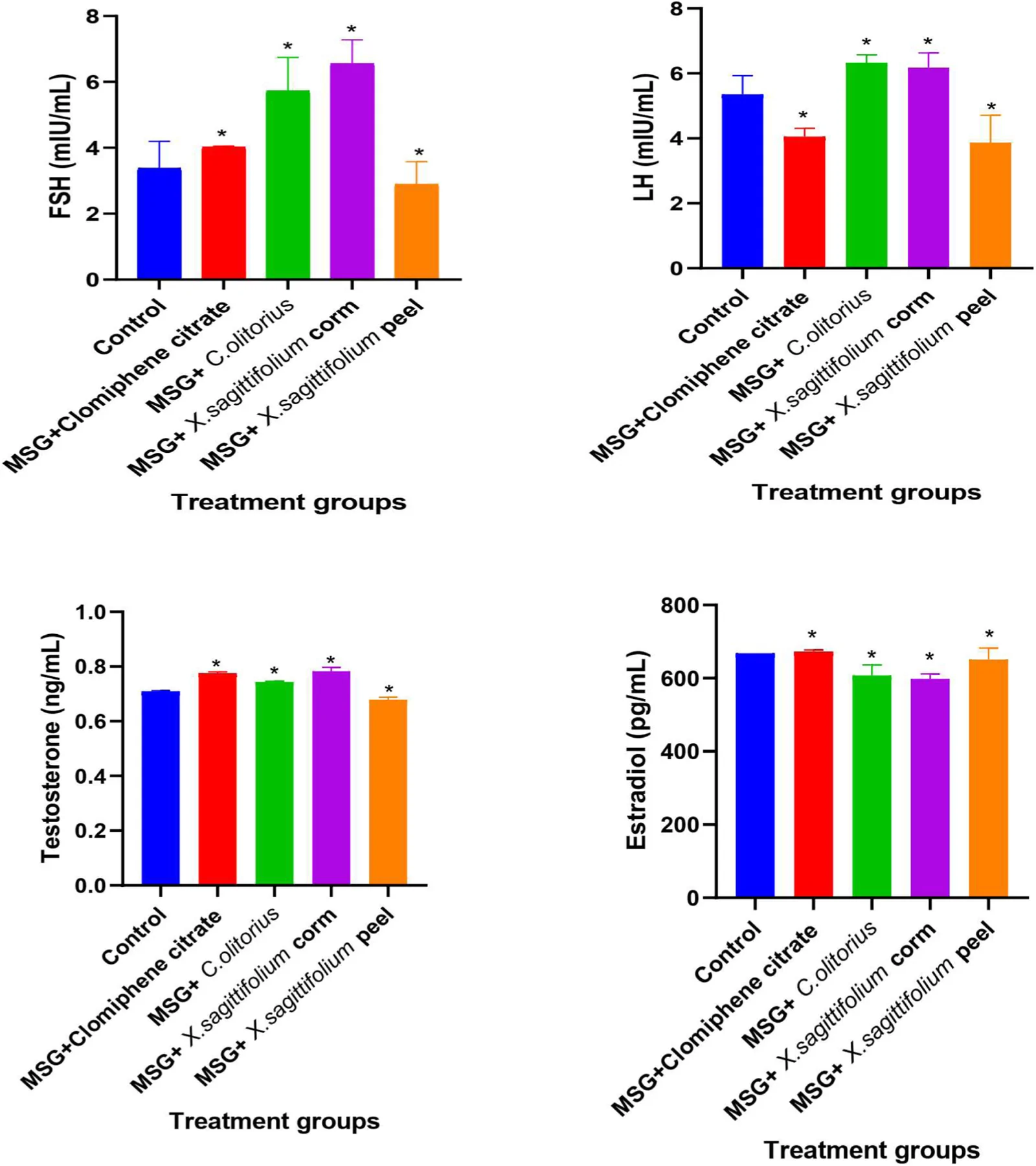
Effect of *C. olitorius*, *X. sagittifolium* corm, and *X. sagittifolium* peel treatments on FSH, LH, Testosterone, and Estradiol levels. * means there is a significant difference compared to control.

**FIGURE 5:**
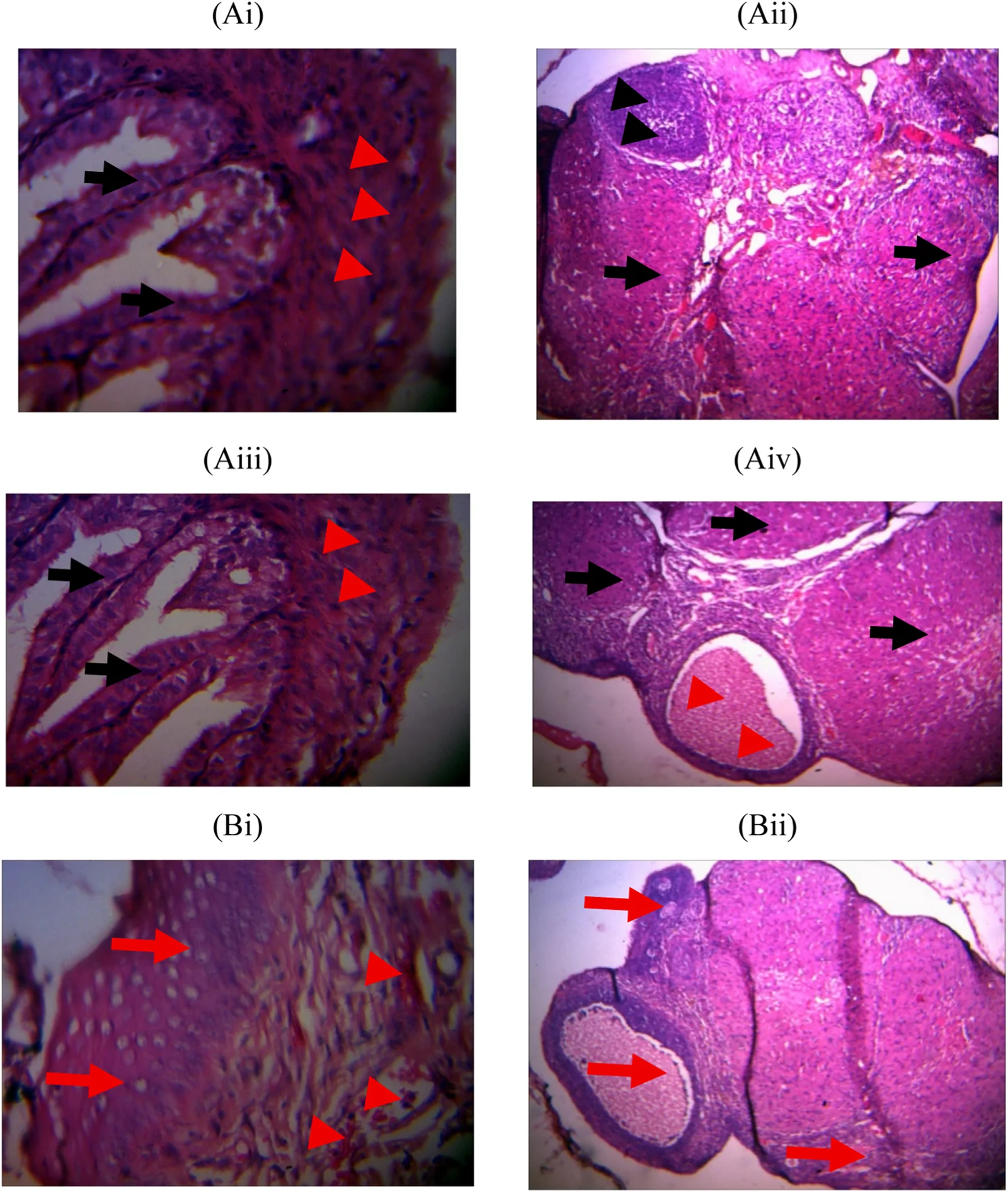
Photomicrographs of the cervix (i and iii) and ovary (ii and iv) of experimental rats stained with hematoxylin and eosin (H&E) (Mag. ×40). Group A (Ai–Aiii) represents the control group, while Bi and Bii represent sections from the MSG + clomiphene citrate–treated group. Ovarian sections show the Graafian follicle (red arrow)**,** the atretic follicle (black arrow), and antral follicle with oocyte (arrowhead)**.** Cervical sections show the endometrial epithelial lining (red arrow) and normal endometrial gland (black arrow)**.**

**FIGURE 6:**
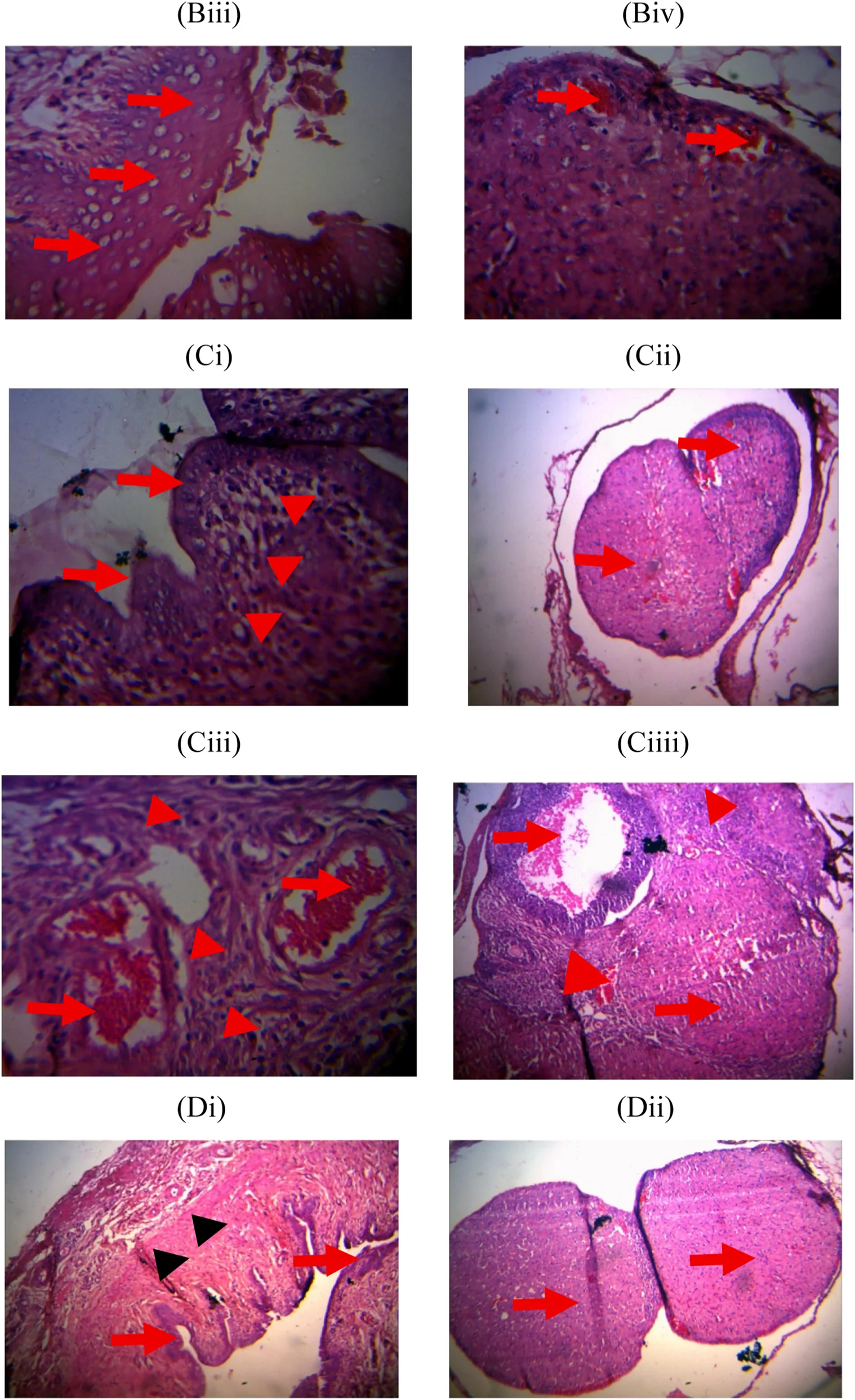
Photomicrographs of the cervix (i and iii) and ovary (ii and iv) of experimental rats stained with hematoxylin and eosin (H&E) (Mag. ×40). Sections shown include Biii–Biiii from the MSG + clomiphene citrate-treated group**,** Ci–Ciiii from the MSG + *Corchorus olitorius-treated* group**,** and Di and Dii from the MSG + *Xanthosoma sagittifolium* corm-treated group. Ovarian sections show the Graafian follicle (red arrow) and antral follicle with oocyte (arrowhead). Cervical sections show the endometrial epithelial lining (red arrow) and normal endometrial gland (black arrow)**.**

**FIGURE 7:**
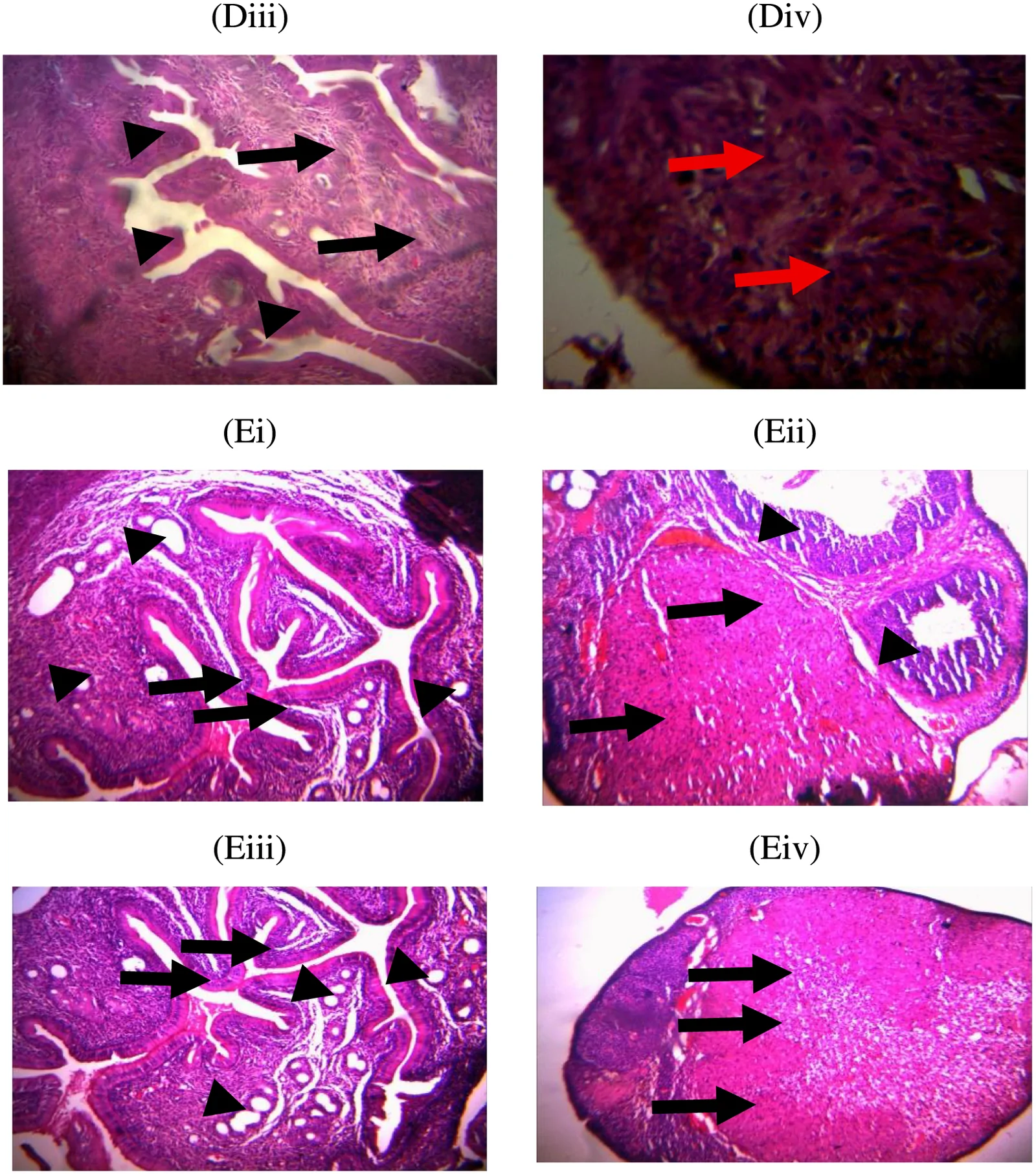
Photomicrographs of the cervix (i and iii) and ovary (ii and iv) of experimental rats stained with hematoxylin and eosin (H&E) (Mag. ×40). Sections shown include Diii–Div from the MSG + *Xanthosoma sagittifolium* corm-treated group and Ei–Eiii from the MSG + *Xanthosoma sagittifolium* peel-treated group. In the ovary, the Graafian follicle (red arrow)**,** atretic follicle (black arrow), and antral follicle with oocyte (arrowhead) are identified. In the cervix, the endometrial epithelial lining (arrowhead) and normal endometrial gland (black arrow) are observed.

The concentrations of follicle-stimulating hormone (FSH), luteinizing hormone (LH), testosterone, and estradiol following monosodium glutamate (MSG) induction and subsequent treatments are presented in Figure 4. Animals that received only distilled water (normal control) maintained baseline levels of all assessed hormones. In contrast, MSG administration resulted in significant alterations in FSH, LH, testosterone, and estradiol concentrations. Treatment with the standard drug, clomiphene citrate, produced significant changes in all evaluated hormones when compared with the normal control. Similarly, administration of *Corchorus olitorius*, *Xanthosoma sagittifolium* corm, and *Xanthosoma sagittifolium* peel following MSG exposure also resulted in significant differences in hormonal concentrations relative to the control. Overall, the results demonstrate that both the standard drug and the plant treatments significantly modulated MSG-induced hormonal alterations across all assessed reproductive hormones. Figures 5–7 show the ovaries’ and cervixes’ tissues under the microscope for histopathological changes or tissue alterations that could have been induced by the administration of monosodium glutamate (MSG).

### 3.3.2 Phytochemistry

**FIGURE 8:**
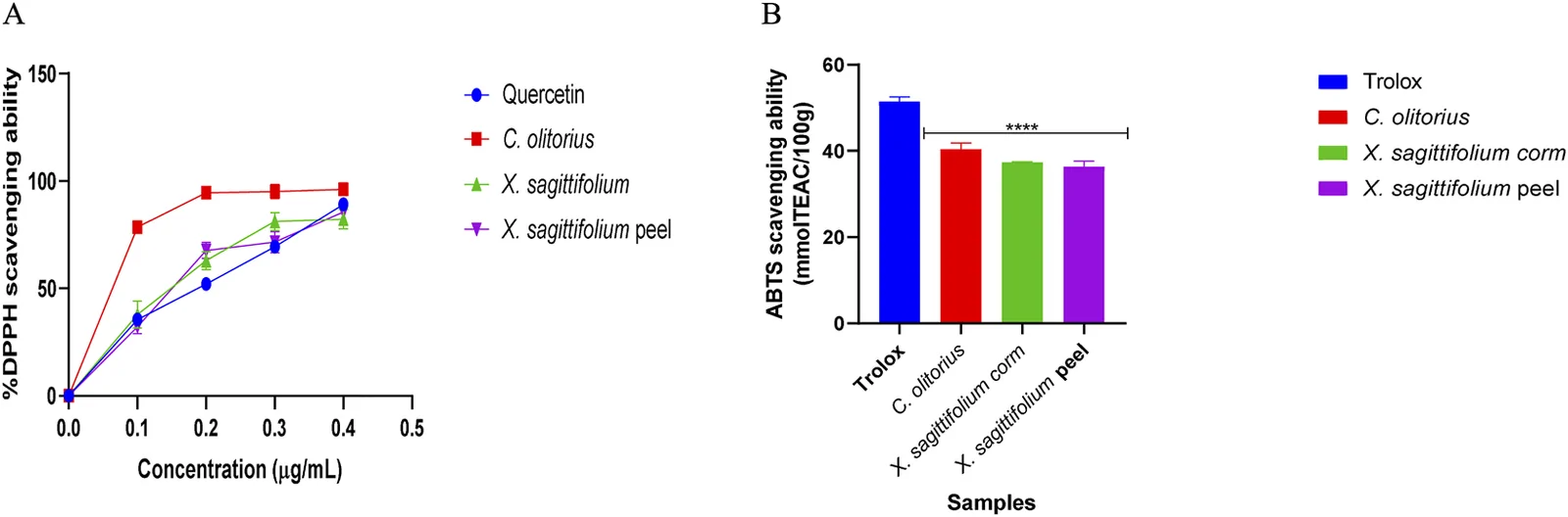
Percentage DPPH radical scavenging **(A)** and ABTS scavenging abilities **(B)** of *C. olitorus*, *X. sagittifolium* corm, *X. sagittifolium* peel and Trolox (standard). Bars represent ± SD (n = 3). Values are statistically different at ****p < 0.001 vs. Trolox.

**FIGURE 9:**
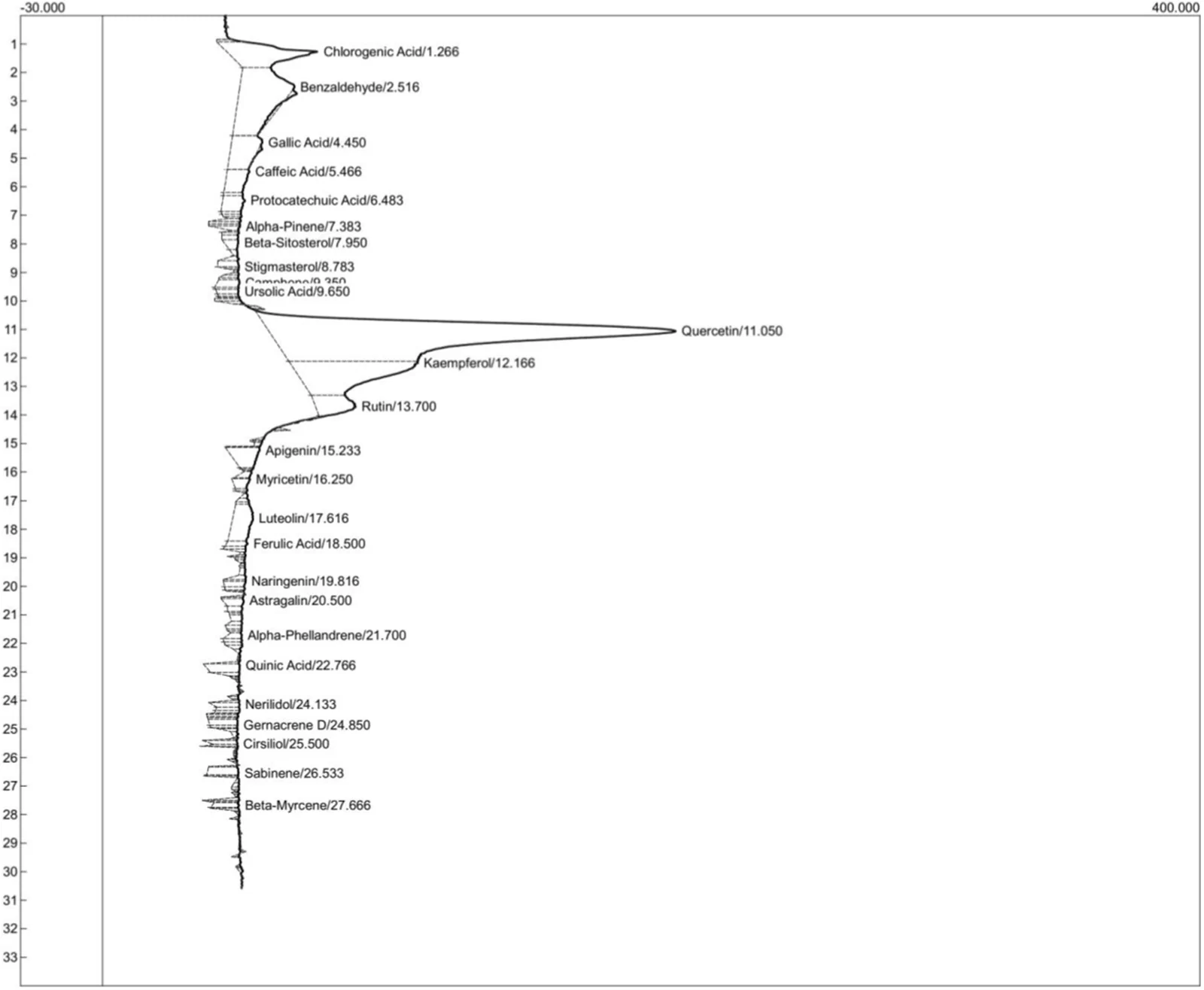
HPLC Chromatogram of *C. olitorius*.

**FIGURE 10:**
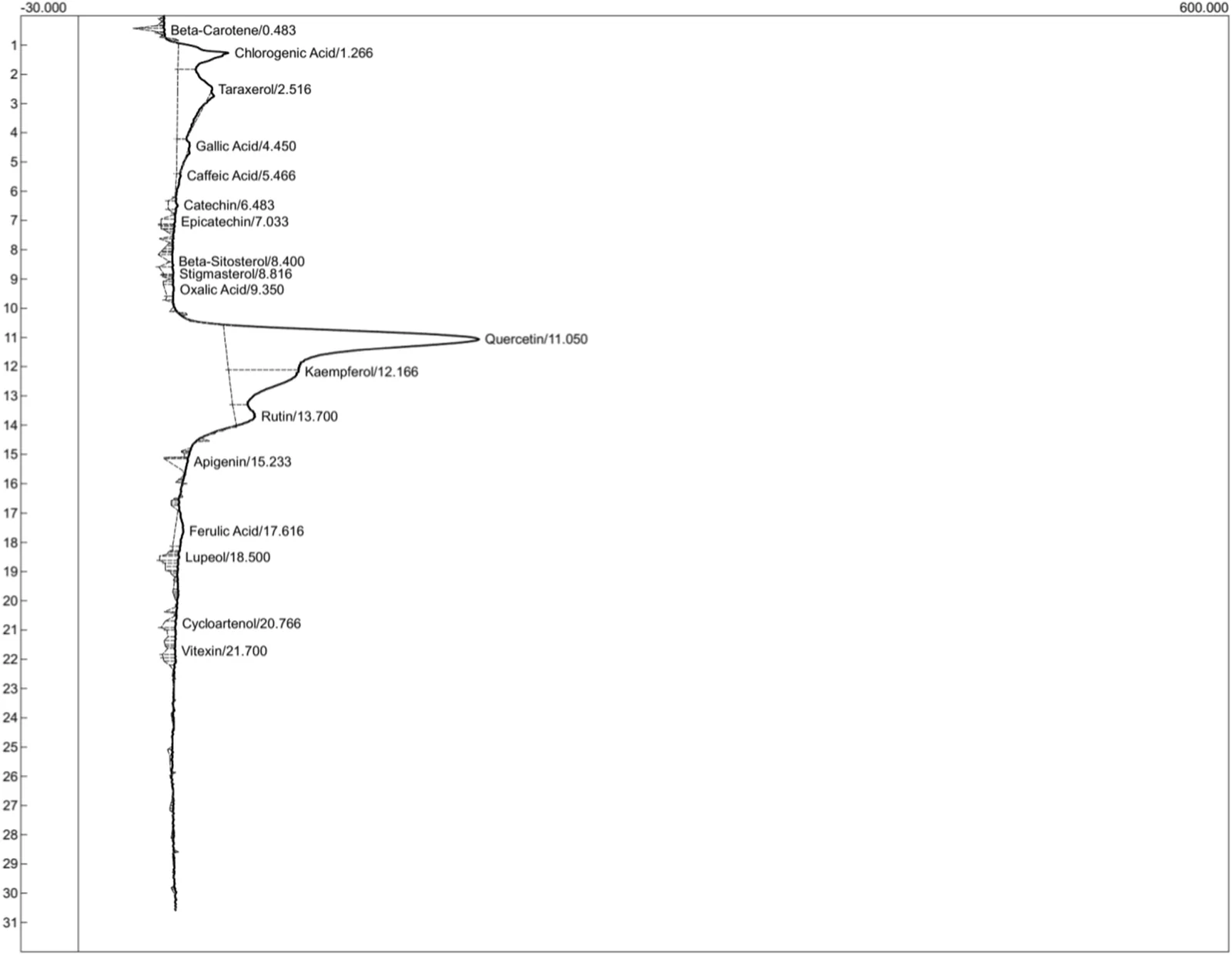
HPLC Chromatogram of *Xanthosoma sagittifolium* corm.

**FIGURE 11:**
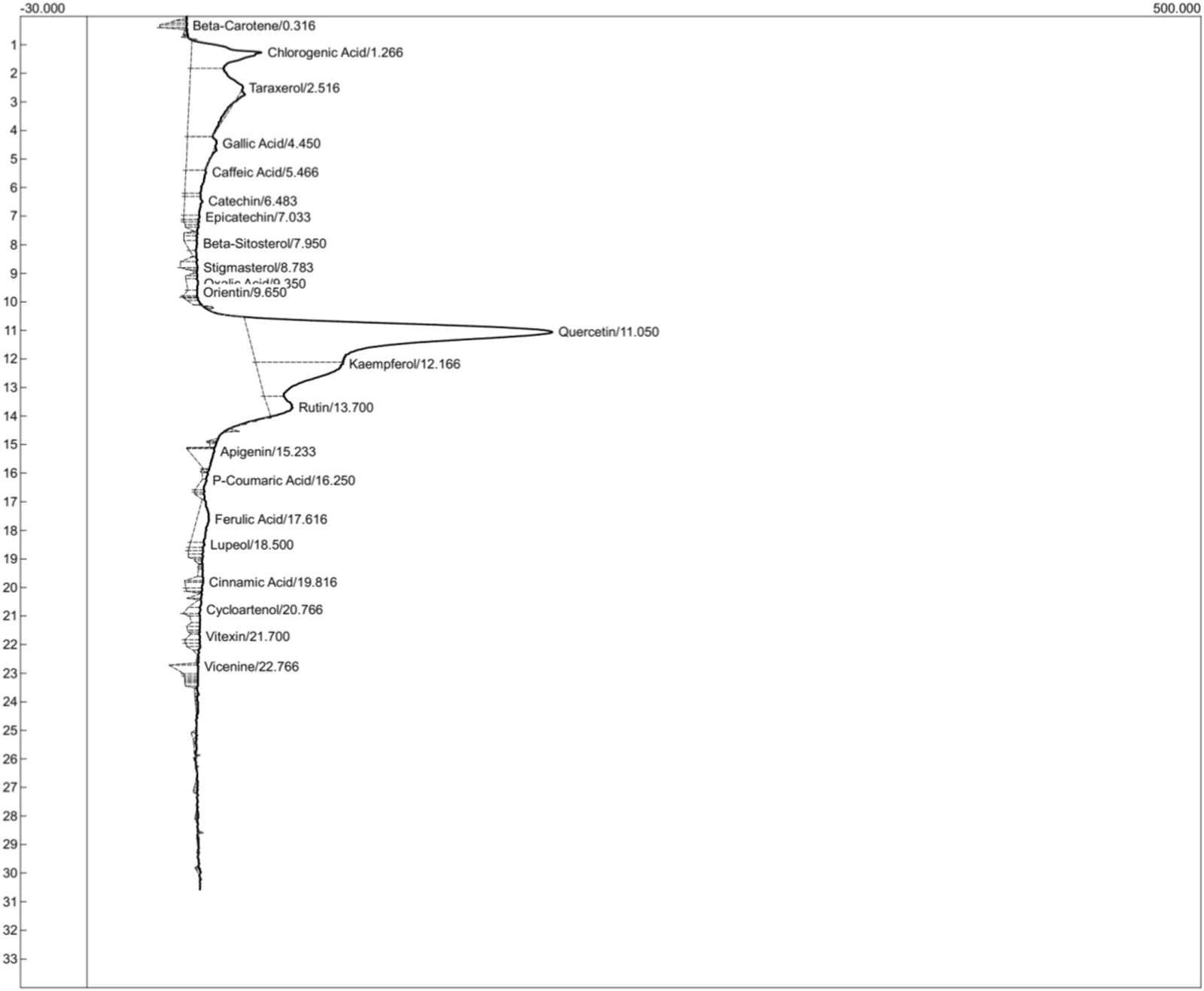
HPLC Chromatogram of *Xanthosoma sagittifolium* peel.

The DPPH radical scavenging activities presented in Figure 8A indicate that *Corchorus olitorius* exhibited stronger radical scavenging activity than *Xanthosoma sagittifolium* and its peel. The activities of *X. sagittifolium* and *X. sagittifolium* peel were comparable to each other but lower than that of *C. olitorius*, while differences relative to the standard antioxidant were also observed. In the ABTS radical scavenging assay (Figure 8B), *C. olitorius* again demonstrated higher antioxidant capacity compared with *X. sagittifolium* and its peel. The peel extract showed slightly greater activity than the flesh, indicating a marginal enrichment of radical scavenging constituents in the peel. In *Corchorus olitorius* (Figure 9; Table 5), flavonoids dominate the profile, with quercetin (45.07%) and kaempferol (11.85%) being the most abundant. Other notable compounds include chlorogenic acid (6.07%), gallic acid (3.87%), and rutin (3.00%), all of which are recognized for their antioxidant properties. Minor constituents such as phytosterols (beta-sitosterol and stigmasterol), monoterpenes (alpha-pinene, camphene, and sabinene), and triterpenoids (ursolic acid) further enrich its phytochemical diversity, though at lower percentages. In contrast, *Xanthosoma sagittifolium* corm (Figure 10) exhibits a relatively modest phytochemical profile, with quercetin (0.524%) and kaempferol (0.126%) as the leading flavonoids. Phenolic acids such as chlorogenic acid (0.061%) and gallic acid (0.026%) are present in trace amounts (Table 6), alongside triterpenoids like taraxerol (0.138%). This indicates a lower concentration of bioactive compounds compared to *C. olitorius*. However, the peel of *X. sagittifolium* (Figure 11) shows a strikingly different composition, with quercetin (43.418%) and kaempferol (10.699%) dominating, alongside high levels of taraxerol (16.265%). Chlorogenic acid (6.689%) and gallic acid (5.046%) also feature prominently (Table 7), suggesting that the peel is a richer source of antioxidants and triterpenoids than the corm.

**TABLE 5:** Compounds detected by HPLC and their percentage composition for *Corchorus olitorius*.

Retention time	Compound	Molecular formula	Molecular weight (g/mol)	Class	Percentage composition (%)
1.266	Chlorogenic acid	**C**_**16**_ **H**_**1**_ **8O**_**9**_	354.31	Phenolic acid	6.07
2.516	Benzaldehyde	C_6_H_5_CHO	106.12	Aromatic aldehyde	0.0012
4.450	Gallic acid	C_7_H_6_O_5_	107.12	Phenolic acid	3.87
5.466	Caffeic acid	C_9_H_8_O_4_	180.16	Polyphenol	2.00
6.483	Protocatechuic acid	C7H6O4	154.12	Phenolic acid	1.39
7.383	Alpha-pinene	C_10_H_16_	136.24	Bicyclic monoterpenoid	0.39
7.950	Beta-sitosterol	C_29_H_50_O	414.7	Phytosterol	0.0005
8.783	Stigmasterol	C_29_H_48_O	412.7	Phytosterol	0.54
9.350	Camphene	C_10_H_16_	136.23	Bicyclic monoterpenoid	0.73
9.650	Ursolic acid	C_30_H_48_O_3_	456.71	Triterpenoid	0.44
11.050	Quercetin	C_15_H_10_O_7_	302.24	Flavonoid	45.07
12.166	Kaempferol	C_15_H_10_O_6_	286.24	Flavonoid	11.85
13.700	Rutin	C_27_H_30_O_16_	610.52	Flavonol	3.00
15.233	Apigenin	C_15_H_10_O_5_	270.24	Flavonoid	2.14
16.250	Myricetin	C_15_H_10_O_8_	318.24	Flavonoid	0.0001
17.616	Luteolin	C_15_H_10_O_6_	286.24	Flavonoid	2.99
18.500	Ferulic acid	C_10_H_10_O_4_	194.18	Phenolic acid	0.46
19.816	Naringenin	C_15_H_12_O_5_	272.25	Flavanone	0.52
20.500	Astragalin	C_21_H_20_O_11_	448.38	Flavonoid	0.62
21.700	Alpha-phellandrene	C_10_H_16_	136.23	Cyclic monoterpene	0.36
22.766	Quinic acid	C_7_H_12_O_6_	192.17	Cyclitol	1.21
24.133	Nerilidol	C_15_H_26_O	222.37	Sesquiterpene alcohol	0.55
24.850	Gernacrene D	C_15_H_24_	204.35	Sesquiterpene	0.76
25.500	Cirsiliol	C_17_H_14_O_7_	330.29	Flavonoid	0.48
26.533	Sabinene	C_10_H_16_	136.23	Bicyclic monoterpene	1.00
27.666	Beta-myrcene	C_10_H_16_	136.23	Acyclic monoterpene	0.53

**TABLE 6:** Compounds detected by HPLC and their percentage composition for *Xanthosoma sagittifolium* corm.

Retention	Compound	Molecular formular	Molecular weight (g/mol)	Class	Percentage Composition (%)
0.483	Beta-carotene	C_40_H_56_	536.9	Carotenoid	0.005
1.266	Chlorogenic acid	C_16_H_18_O_9_	354.30	Phenolic acid	0.061
2.516	Taraxerol	C_30_H_50_O	426.7	Pentacyclic triterpenoid	0.138
4.450	Gallic acid	C_7_H_6_O_5_	170.12	Phenolic acid	0.026
5.466	Caffeic acid	C_9_H_8_O_4_	180.16	Polyphenol	0.005
6.483	Catechin	C_15_H_14_O_6_	290.271	Flavonoid	0.006
7.033	Epicatechin	C_22_H_18_O_10_	442.373	Flavonoid	0.006
8.400	Beta-sitosterol	C_29_H_50_O	414.718	Phytosterol	0.005
8.816	Stigmasterol	C_29_H_48_O	412.702	Phytosterol	0.007
9.350	Oxalic acid	C_2_H_2_O_4_	126.065	Organic acid	0.008
11.050	Quercetin	C_15_H_10_O_7_	302.236	Flavonoid	0.524
12.166	Kaempferol	C_15_H_10_O_6_	286.23	Flavonoid	0.126
13.700	Rutin	C_27_H_30_O_16_	610.521	Flavonoid	0.028
15.233	Apigenin	C_15_H_10_O_5_	270.240	Flavonoid	0.014
17.616	Ferulic acid	C_10_H_10_O_4_	194.186	Phenolic phytochemical	0.019
18.500	Lupeol	C_30_H_50_O	426.729	Triterpenoid	0.007
20.766	Cycloartenol	C_30_H_50_O	426.72	Triterpenoid	0.007
21.700	Vitexin	C_21_H_20_O_10_	432.38	Flavonoid	0.005

**TABLE 7:** Compounds detected by HPLC and their percentage composition for *Xanthosoma sagittifolium* peel.

Retention	Compound	Molecular formular	Molecular weight (g/mol)	Class	Percentage Composition (%)
0.316	Beta-carotene	C_40_H_56_	536.9	Carotenoid	0.458
1.266	Chlorogenic acid	C_16_H_18_O_9_	354.30	Phenolic acid	6.689
2.516	Taraxerol	C_30_H_50_O	426.7	Pentacyclic triterpenoid	16.265
4.450	Gallic acid	C_7_H_6_O_5_	170.12	Phenolic acid	5.046
5.466	Caffeic acid	C_9_H_8_O_4_	180.16	Polyphenol	2.435
6.483	Catechin	C_15_H_14_O_6_	290.271	Flavonoid	1.794
7.033	Epicatechin	C_22_H_18_O_10_	442.373	Flavonoid	0.395
7.950	Beta-sitosterol	C_29_H_50_O	414.718	Phytosteroid	0.585
8.783	Stigmasterol	C_29_H_48_O	412.702	Phytosteroid	0.605
9.350	Oxalic acid	C_2_H_2_O_4_	126.065	Organic acid	0.724
9.650	Orientin	C_21_H_20_O_11_	448.38	Flavonoid	0.388
11.050	Quercetin	C_15_H_10_O_7_	302.236	Flavonoid	43.418
12.166	Kaempferol	C_15_H_10_O_6_	286.23	Flavonoid	10.699
13.700	Rutin	C_27_H_30_O_16_	610.521	Flavonoid	2.368
15.233	Apigenin	C_15_H_10_O_5_	270.240	Flavonoid	2.062
16.250	P-coumaric acid	C_9_H_8_O_3_	164.160	Phenolic acid	0.405
17.616	Ferulic acid	C_10_H_10_O_4_	194.186	Phenolic acid	2.620
18.500	Lupeol	C_30_H_50_O	426.729	Triterpenoid	0.436
19.816	Cinnamic acid	C_9_H_8_O_2_	148.161	Carboxylic acid	0.576
20.766	Cycloartenol	C_30_H_50_O	426.72	Triterpenoid	0.527
21.700	Vitexin	C_21_H_20_O_10_	432.38	Flavonoid	0.397
22.766	Vicenine	C_26_H_28_O	564.5	Flavonoid	1.109
16.250	P-coumaric acid	C_9_H_8_O_3_	164.160	Phenolic acid	0.405
17.616	Ferulic acid	C_10_H_10_O_4_	194.186	Phenolic acid	2.620
18.500	Lupeol	C_30_H_50_O	426.729	Triterpenoid	0.436
19.816	Cinnamic acid	C_9_H_8_O_2_	148.161	Carboxylic acid	0.576
20.766	Cycloartenol	C_30_H_50_O	426.72	Triterpenoid	0.527
21.700	Vitexin	C_21_H_20_O_10_	432.38	Flavonoid	0.397
22.766	Vicenine	C_26_H_28_O	564.5	Flavonoid	1.109

## 4 Discussion

### 4.1 *In vitro* antioxidant, antidiabetic, and inhibition of cholinesterase

Uterine leiomyoma is a multifactorial disorder characterized by excessive smooth muscle cell proliferation, extracellular matrix (ECM) accumulation, oxidative stress, inflammation, and dysregulated cellular signaling (Farris et al., 2019; Sohn et al., 2018). Increasing evidence indicates that oxidative stress and enzyme-mediated metabolic and inflammatory pathways play crucial roles in fibroid initiation and progression, largely through reactive oxygen species (ROS)-induced cellular proliferation and fibrotic remodeling (Pinto, 2024; Ogunlakin et al., 2025a). In this study, the *in vitro* antioxidant and enzyme inhibitory activities of *Corchorus olitorius*, *Xanthosoma sagittifolium* (corm), and *Xanthosoma sagittifolium* peel to provide mechanistic insight into their potential relevance in the management of uterine leiomyoma. Oxidative stress has been implicated in the pathogenesis of uterine leiomyoma through enhanced production of ROS, which stimulate smooth muscle cell proliferation and excessive deposition of ECM components such as collagen and fibronectin (Farris et al., 2019; Pinto, 2024). The strong antioxidant activities observed in this study therefore suggest a potential protective role against fibroid development by limiting oxidative damage. The free radical scavenging and reducing activities demonstrated by *Cochorus olitorius* indicate a strong ability to neutralize reactive oxygen species (ROS). Such activity is biologically relevant, as excessive ROS production has been shown to upregulate growth factors and cytokines that drive leiomyoma cell proliferation and fibrotic remodeling (Sohn et al., 2018). By donating electrons or hydrogen atoms, antioxidants may interrupt oxidative chain reactions and limit oxidative stress–induced cellular signaling. The metal chelating activity observed particularly in *Xanthosoma sagittifolium* peel is also significant in the context of fibroid pathology. Transition metals such as iron catalyze the formation of highly reactive radicals through Fenton-type reactions, thereby exacerbating oxidative injury and fibrotic tissue remodeling (Ogunlakin et al., 2025b). The ability of the peel extract to chelate metal ions suggests a complementary antioxidant mechanism that may reduce iron-mediated oxidative stress within uterine tissue. Nitric oxide scavenging activity was comparatively weaker across the plant extracts. Nitric oxide is involved in inflammatory and vascular signaling, and its dysregulation has been implicated in fibroid-associated inflammation (Aninye et al., 2025). The limited nitric oxide scavenging observed suggests that the antioxidant effects of the extracts are more strongly mediated through direct radical scavenging, reducing power, and metal chelation rather than modulation of nitric oxide pathways. C. olitorius’s activity aligns with this more comprehensive antioxidant profile. It is well known that medicinal plants like Camellia sinensis and Curcuma longa inhibit ROS-driven signalling pathways, which lessens fibrotic remodelling (Martínez-Iglesias et al., 2025; Gore et al., 2026). Both Hibiscus sabdariffa and Moringa oleifera have been shown to have chelating properties that lessen oxidative stress caused by iron (Al-Hashimi, 2012; Oboh et al., 2015). The activity of the peel extract indicates that by focusing on metal-induced radical production, it may supplement ROS scavengers. Stronger NO scavenging and anti-inflammatory properties are exhibited by plants like *Zingiber officinale* Roscoe (Zingiberaceae) and *Allium sativum* L. (Amaryllidaceae) (Mustafa et al., 2019; Shang et al., 2019). C. olitorius and X. sagittifolium seem to be less effective in reducing NO-mediated inflammation in comparison to these. Overall, the antioxidant findings suggest that *Cochorus olitorius* may be particularly effective in counteracting ROS-driven cellular proliferation, while *Xanthosoma sagittifolium* peel may contribute to reducing metal-induced oxidative damage. These complementary actions are relevant to limiting oxidative stress–associated leiomyoma progression.

Enzyme-mediated metabolic and inflammatory pathways have been increasingly implicated in uterine leiomyoma pathophysiology. Dysregulated carbohydrate metabolism and insulin resistance have been associated with increased fibroid risk, potentially through enhanced growth factor signaling and hormonal imbalance (Borah et al., 2013). Inhibition of carbohydrate-hydrolyzing enzymes such as α-amylase and α-glucosidase may therefore indirectly modulate metabolic conditions that favor fibroid growth. The observed inhibition of α-amylase and α-glucosidase by the plant extracts suggests their potential to regulate postprandial glucose levels and reduce metabolic stress. Improved metabolic control may attenuate insulin-mediated proliferative signaling pathways that contribute to leiomyoma enlargement (Farris et al., 2019). Acetylcholinesterase inhibition is also relevant beyond its classical neurological role. Acetylcholine and cholinergic signaling have been linked to inflammatory modulation and cellular proliferation. Inhibition of acetylcholinesterase may enhance acetylcholine availability, thereby exerting anti-inflammatory effects that could suppress inflammatory processes involved in leiomyoma growth (Sohn et al., 2018). Chronic inflammation is known to promote ECM deposition and fibrotic progression in uterine leiomyomas (Pinto, 2024). Trigonella foenum-graecum and Momordica charantia are well-known inhibitors of enzymes that hydrolyse carbohydrates, enhance glycemic management, and lower insulin resistance (Dhull et al., 2021; Oyelere et al., 2022; Richter et al., 2023; Katipearachchi et al., 2025). This is consistent with the activity seen in Xanthosoma sagittifolium and Corchorus olitorius, supporting their significance in metabolic regulation associated with leiomyoma risk. *Rosmarinus officinalis* L. and *Salvia officinalis* L. are recognised acetylcholinesterase inhibitors with anti-inflammatory properties (Lopresti, 2017; Kosmopoulouv et al., 2024). Similar to the mechanism suggested in this study, their activity has been connected to decreased cytokine production and ECM deposition (Roohbakhsh et al., 2020; Tričković et al., 2026). *Zingiber officinale* and *Curcuma longa* L. (Zingiberaceae) are well known for their anti-inflammatory enzyme regulation, which lowers fibrotic signalling and NF-κB activation. The combined inhibition of metabolic and inflammatory enzymes observed in this study indicates that the plant extracts possess multitarget bioactivity. Such multitarget actions are advantageous in managing complex disorders like uterine leiomyoma, where oxidative stress, inflammation, metabolic dysregulation, and fibrosis coexist.

#### 4.1.1 *In vivo* hormonal analysis

The present study demonstrated that monosodium glutamate (MSG) administration significantly disrupted reproductive hormonal balance, as evidenced by alterations in follicle-stimulating hormone (FSH), luteinizing hormone (LH), testosterone, and estradiol levels. These findings align with previous reports indicating that MSG exposure interferes with hypothalamic–pituitary–gonadal (HPG) axis regulation through neuroendocrine toxicity and oxidative stress, leading to impaired gonadotropin release and steroidogenesis (Eweka et al., 2011; Igwebuike et al., 2011). The maintenance of normal hormonal levels in the distilled water–treated control animals confirms physiological endocrine homeostasis in the absence of MSG-induced stress. Treatment with clomiphene citrate resulted in significant modulation of all assessed hormones, validating its role as a standard therapeutic agent in restoring gonadotropin secretion and normalizing estrogen–androgen balance. Clomiphene citrate acts primarily as a selective estrogen receptor modulator, enhancing pituitary release of FSH and LH by blocking estrogenic negative feedback at the hypothalamic level, which subsequently improves gonadal hormone regulation (Farris et al., 2019; Sohn et al., 2018). The response observed in the standard-treated group therefore confirms the sensitivity and reliability of the experimental model. Administration of *Corchorus olitorius* following MSG exposure produced significant changes in FSH, LH, testosterone, and estradiol levels, suggesting a modulatory influence on reproductive endocrine function. This effect may be attributed to the plant’s rich phytochemical composition, particularly flavonoids, phenolic compounds, and antioxidants, which have been shown to protect endocrine tissues from oxidative damage and improve hormonal signaling pathways (Arip et al., 2022; Ogunlakin et al., 2025b). The ability of *C. olitorius* to influence both gonadotropins and sex steroids supports its potential role in mitigating hormone-related uterine pathologies. Similarly, treatment with *Xanthosoma sagittifolium* corm and peel significantly altered hormonal concentrations compared with the control, indicating endocrine-modulating activity. Although limited studies exist on *X. sagittifolium* in reproductive disorders, its reported antioxidant and anti-inflammatory properties may contribute to the restoration of hormonal balance by reducing MSG-induced oxidative stress and improving ovarian steroidogenesis (Ajayi et al., 2025). The differential responses observed between the corm and peel treatments further suggest variation in bioactive compound distribution within the plant, which may influence hormonal outcomes. Estradiol modulation observed across the plant-treated groups is particularly relevant in the context of uterine leiomyoma, a hormone-dependent condition characterized by estrogen-driven smooth muscle proliferation and extracellular matrix accumulation (Pinto, 2024; Borah et al., 2013). The ability of *Corchorus olitorius* and *Xanthosoma sagittifolium* extracts to regulate estradiol levels suggests a possible mechanism through which these plants may exert antifibrotic or anti-leiomyoma effects. Additionally, the observed regulation of FSH and LH implies broader normalization of the HPG axis, which is essential for maintaining reproductive endocrine balance.

By shielding hypothalamus and pituitary tissues from oxidative damage, *Withania somnifera* Dunal (Solanaceae) and *Panax ginseng* Meyer (Araliaceae) restored gonadotropin secretion and normalise sex steroid levels (Wiciński et al., 2023; El-Shimi et al., 2024). This is similar to the modulatory actions of Xanthosoma sagittifolium and Corchorus olitorius*. Glycyrrhiza glabra* L. (Fabaceae) and *Vitex agnus-castus* L. (Lamiaceae) are known to reduce oestrogen dominance by regulating the balance of progesterone and oestrogen (Alamoudi and Bakrshoom, 2021; Kandeel et al., 2025). These phytoestrogenic and hormone-modulating plants are consistent with the regulation of oestradiol seen in C. olitorius and X. sagittifolium. It has been demonstrated that *Tribulus terrestris* L. (Zygophyllaceae) and *Lepidium meyenii* Walpers (Brassicaceae) improve reproductive endocrine balance by increasing LH and FSH secretion (Kasprzak et al., 2018; Saeed et al., 2024). These gonadotropin-supporting effects of these plants are consistent with the action of C. olitorius and X. sagittifolium. Both Camellia sinensis and Curcuma longa stabilise hormone output by shielding endocrine tissues from oxidative stress (Sánchez et al., 2020; Wang et al., 2024). These antioxidant-rich plants’ efficacy aligns with the mechanism of C. olitorius and X. sagittifolium. The findings of this study indicate that *Corchorus olitorius* and *Xanthosoma sagittifolium* (corm and peel) possess significant hormonal modulatory properties comparable to the standard drug. These effects may be mediated through antioxidant activity, endocrine tissue protection, and regulation of gonadotropin and steroid hormone secretion. The hormonal regulation observed provides mechanistic support for the potential use of these plants in the management of hormonally driven reproductive disorders, including uterine leiomyoma, and complements existing evidence on the therapeutic relevance of botanicals in fibroid management (Ogunlakin et al., 2025a; Aninye et al., 2025).

#### 4.1.2 Histopathological examination of uterus and cervix

Histopathological examination of the cervix and ovary was conducted to evaluate the effects of monosodium glutamate (MSG) and the modulatory roles of clomiphene citrate, *Corchorus olitorius*, and *Xanthosoma sagittifolium* extracts. These tissues are highly susceptible to oxidative stress, hormonal imbalance, and fibrotic remodeling, which are key processes implicated in uterine leiomyoma development (Farris et al., 2019; Pinto, 2024). The control group showed preserved cervical and ovarian architecture, confirming normal tissue integrity and physiological follicular development. MSG-treated rats receiving clomiphene citrate exhibited cervical fibrous connective tissue accumulation and vascular congestion, indicating persistent fibrotic remodeling despite treatment. Although ovarian sections showed improved follicular maturation, clomiphene citrate appeared to provide limited protection against MSG-induced oxidative and structural tissue damage, likely due to its primary action on hormonal regulation rather than antifibrotic pathways. Treatment with *Corchorus olitorius* resulted in relatively improved epithelial organization of cervical tissues compared with the standard drug group, suggesting partial attenuation of oxidative damage. However, fibrotic changes and ovarian follicular immaturity persisted, indicating that while the extract may reduce tissue injury, its effect on restoring folliculogenesis was limited at the administered dose. Rats treated with *Xanthosoma sagittifolium* corm extract showed marked cervical fibroplasia and incomplete ovarian follicular maturation, reflecting moderate protective activity. In contrast, the corm peel extract demonstrated the most preserved cervical architecture with minimal fibrosis and restored ovarian folliculogenesis, including the presence of Graafian follicles.

Through antioxidant and anti-inflammatory mechanisms, *Nigella sativa* L. (Ranunculaceae) and *Ocimum gratissimum* L. (Lamiaceae) have shown protective effects on reproductive organs, reducing fibrosis and promoting folliculogenesis (Imosemi, 2020; Ouies et al., 2021). This is consistent with X. sagittifolium peel’s higher restorative action. *Vernonia amygdalina* Del. (Asteraceae) and *Phyllanthus niruri* L. (Phyllanthaceae) are well-known for their antioxidant and antifibrotic properties, which reduce oxidative damage and collagen buildup in reproductive organs (Kaur et al., 2017; Kabir et al., 2026). X. sagittifolium peel’s mechanism substantially resembles that of these plants, indicating a similar antifibrotic function. Recently, it has been found that *Crassocephalum crepidiodes* Benth S. Moore (Asteraceae) enhanced gonadal function and ovarian follicular development (Ogunlakin et al., 2025a). These plants that support reproduction are similar to the folliculogenic properties of X. sagittifolium corm. This superior effect of *Xanthosoma sagittifolium* corm may be attributed to the strong antioxidant and metal-chelating properties of the peel, which likely reduced oxidative stress and supported normal tissue remodeling. The histopathological findings indicate that MSG induces significant reproductive tissue damage characterized by fibrosis and disrupted folliculogenesis. Among the treatments evaluated, *X. sagittifolium* corm peel exhibited the greatest protective and restorative effects, highlighting its potential relevance in modulating fibroid-associated tissue pathology.

### 4.2 Phytochemicals detected and fibroid management

Compared with other medicinal plants, the DPPH and ABTS scavenging values of *C. olitorius* and *X. sagittifolium* are moderate, aligning with typical results found in leafy and tuberous medicinal plants such as *Moringa oleifera* Lam. (Moringaceae) or *Curcuma longa* (Siddhuraju and Becker, 2003; Tanvir et al., 2017; Xu et al., 2019; Roytrakul et al., 2024). However, these results stem solely from chemical assays that measure radical-quenching potential, not biological efficacy. They indicate phytochemical presence rather than therapeutic potency. Thus, these findings belong strictly to the phytochemical-analytical domain, emphasizing compositional insight rather than medicinal efficacy. The HPLC analysis revealed that *Xanthosoma sagittifolium* and *Corchorus olitorius* contain diverse bioactive compounds predominantly belonging to flavonoids, phenolic acids, phytosterols, carotenoids, and triterpenoids. The presence of these phytochemical classes suggests that the biological activities observed in this study are likely mediated through synergistic interactions among multiple constituents rather than a single active compound, which is particularly relevant for a multifactorial condition such as uterine leiomyoma characterized by oxidative stress, inflammation, hormonal dysregulation, and excessive extracellular matrix accumulation (Farris et al., 2019; Pinto, 2024).

The phytochemical profile of *Corchorus olitorius* was dominated by flavonoids, with quercetin occurring as the most abundant compound, followed by kaempferol, rutin, apigenin, luteolin, and other minor flavonoids. High flavonoid abundance is indicative of strong antioxidant and antiproliferative potential, as flavonoids are known to scavenge reactive oxygen species, inhibit smooth muscle cell proliferation, and modulate signaling pathways involved in fibroid growth (Sohn et al., 2018; Ogunlakin et al., 2025b). The detection of phenolic acids such as chlorogenic acid, gallic acid, caffeic acid, protocatechuic acid, ferulic acid, and quinic acid further supports the ability of *C. olitorius* to attenuate oxidative stress and inflammatory responses, both of which play central roles in leiomyoma initiation and progression (Borah et al., 2013; Pinto, 2024). Marked differences were observed between *Xanthosoma sagittifolium* peel and corm in terms of phytochemical composition. *Xanthosoma sagittifolium* peel exhibited greater phytochemical diversity and higher compound abundance compared to the corm. Notably, orientin, vitexin, vicenine, p-coumaric acid, cinnamic acid, and oxalic acid were detected in the peel but were absent in the corm. These compounds, particularly the flavonoid glycosides orientin, vitexin, and vicenine, have been associated with antioxidant, anti-inflammatory, and cytoprotective activities, which may enhance the biological effectiveness of the peel extract in modulating fibroid-related pathways (Ogunlakin et al., 2025b).

In addition to the unique compounds present in the peel, shared phytochemicals such as chlorogenic acid, gallic acid, caffeic acid, ferulic acid, quercetin, kaempferol, rutin, and apigenin occurred at higher levels in the peel than in the corm. *Xanthosoma sagittifolium* peel also contained triterpenoids and phytosterols, including lupeol, taraxerol, cycloartenol, β-sitosterol, and stigmasterol, which were absent or detected at very low levels in the corm. These compounds are biologically relevant due to their reported ability to modulate estrogen-related signaling, suppress inflammatory mediators, and reduce fibroblast activation and collagen deposition, processes closely linked to uterine leiomyoma growth (Farris et al., 2019; Pinto, 2024). Conversely, *Xanthosoma sagittifolium* corm exhibited a relatively limited phytochemical profile with lower concentrations of most detected compounds. The absence of several bioactive constituents and the reduced abundance of key flavonoids and phenolic acids suggest a comparatively lower biological potential of the corm. This observation supports existing evidence that plant parts exposed to environmental stress, such as peels, tend to accumulate higher levels of protective secondary metabolites compared to storage tissues (Ogunlakin et al., 2025b). The HPLC findings indicate that *Xanthosoma sagittifolium* peel is rich in phytochemicals compared to the corm, while *Corchorus olitorius* is rich in flavonoids and phenolic acids. The combined presence of antioxidants, phytosterols, and triterpenoids provides a plausible biochemical explanation for the antifibrotic and protective effects observed in this study, thus supporting the potential application of these plant materials in the management of MSG-induced uterine leiomyoma (Sohn et al., 2018; Pinto, 2024).

## 5 Conclusion

This study evaluated indigenous medicinal plants for antifibrotic and protective effects against MSG-induced uterine leiomyoma using *in vitro* and *in vivo* models. MSG exposure caused hormonal disruption and structural distortions in reproductive organs, confirming leiomyoma-like conditions. Plant extracts significantly reversed these changes, preserving tissue architecture and restoring estrogen and progesterone balance. HPLC analysis revealed phenolic compounds and flavonoids with antioxidant, anti-inflammatory, and antifibrotic properties. The extracts also showed strong antioxidant activity, enzyme inhibition, and hormonal regulation. These findings support antioxidants, enzyme inhibitory, and endocrine-modulating properties but have not yet established therapeutic efficacy against uterine leiomyoma in humans. Therefore, further studies on direct uterine tissue evaluation and fibrosis-specific biomarker analysis are hereby recommended. Furthermore, further studies should be conducted to isolate, characterize, and quantify the specific bioactive compounds identified by HPLC that are responsible for the observed antifibrotic and protective effects.

## Data Availability

The original contributions presented in the study are included in the article/supplementary material, further inquiries can be directed to the corresponding authors.
